# Association between Flavonoid Intake and Cognitive Executive Function among African American and White Adults in the Healthy Aging in Neighborhoods of Diversity across the Life Span (HANDLS) Study

**DOI:** 10.3390/nu16091360

**Published:** 2024-04-30

**Authors:** Marie Fanelli Kuczmarski, Sara B. Crawford, Rhonda S. Sebastian, May A. Beydoun, Joseph D. Goldman, Alanna J. Moshfegh, Michele K. Evans, Alan B. Zonderman

**Affiliations:** 1 Laboratory of Epidemiology and Population Sciences, National Institute on Aging, NIH, Baltimore, MD 21224, USA; baydounm@mail.nih.gov (M.A.B.); evansm@grc.nia.nih.gov (M.K.E.); zondermana@gmail.com (A.B.Z.); 2 Food Surveys Research Group, Agricultural Research Service, United States Department of Agriculture, BARC-West, Beltsville, MD 20705, USA; sara.crawford@usda.gov (S.B.C.); rhonda.sebastian@ars.usda.gov (R.S.S.); joseph.goldman@usda.gov (J.D.G.); alanna.moshfegh@usda.gov (A.J.M.)

**Keywords:** flavonoids, cognition, Trail Making Test, African Americans, HANDLS

## Abstract

Healthy dietary patterns rich in flavonoids may benefit cognitive performance over time. Among socioeconomically disadvantaged groups, the association between flavonoid intake and measures of cognition is unclear. This study sought to identify associations between flavonoid intake and cognitive performance among Healthy Aging in Neighborhoods of Diversity across the Life Span (HANDLS) study participants (n = 1947) across three study visits. Flavonoid intakes were assessed via two 24-h dietary recalls. Cognitive performance was assessed via the Trail Making Test (TMT)-A and TMT-B, which provide measures of attention and executive function, respectively. Mixed effects linear regression was used to model TMT scores over three study visits against visit 1 (v1) flavonoid intake, time (years from v1), and the interaction between v1 flavonoid intake and time, capturing both the cross-sectional association between flavonoid intake and time at v1 as well as the longitudinal association between v1 flavonoid intake and the change in TMT scores over time. Prior to adjustment, inverse cross-sectional associations at v1 were observed between (1) anthocyanidin intake and TMT-A scores for the overall sample and (2) total flavonoid, anthocyanidin, flavan-3-ol, flavone, and flavonol intake and TMT-B scores for the overall sample and among White adults. Only the association between anthocyanidin intake and TMT-B at v1 among White adults persisted after adjustment (for demographic characteristics such as age). One possible explanation for the few significant associations is universally low flavonoid intakes resulting from the consumption of an unhealthy dietary pattern.

## 1. Introduction

According to the United States (US) Health and Retirement Study, approximately two out of three American adults experience some level of cognitive impairment (but not dementia) at an average age of approximately 70 years [1]. Cognitive impairment is characterized by trouble remembering, learning new things, concentrating, or making decisions that affect everyday life and can vary from mild to severe [2]. Women have a 71% lifetime risk of experiencing cognitive impairment before death while men have a 61% lifetime risk [1]. There are large disparities in the risk by race, with 72% of Black men and 83% of Black women experiencing impairment, compared to 57% and 66% of White men and women, respectively [1]. Lifetime risk also varies with level of education, as those with less than a high school degree have the greatest risk compared to those with high school or higher educational attainment [1]. However, the mechanisms driving these disparities have not been identified. A challenge for health professionals is to identify strategies to minimize age-related cognitive decline, especially among high-risk populations.

There is evidence, although limited, that flavonoids and/or foods rich in flavonoid compounds have the potential to improve cognition at the preclinical and clinical levels [3,4]. Meta-analyses of randomized controlled trials with multiple populations suggest that flavonoid intake offers protective neurocognitive benefits across a lifespan [5]. Associations differ by food source, which may reflect the bioavailability of flavonoids and flavonoid subclasses [3,5,6,7], the ability of the flavonoid to cross the blood–brain barrier, and the diversity in gut microbiome-generated flavonoid metabolites. These metabolites can also be transported by the vagus nerve and systemic circulation to the brain [8,9,10]. However, findings from both observational and longitudinal studies describing associations between flavonoid intake and cognitive measures have been inconsistent [6,11,12,13,14,15,16]. This may be due to differences in the populations studied, the dietary and/or cognitive assessment methods employed, and/or the other explanatory variables that are included in the models [17].

Impaired executive function and overall cognition are symptoms of major depression, a disease that affects approximately 21 million Americans annually [18]. Flavonoid consumption is associated with a decreased risk of developing depression [19]. There is also evidence that diets rich in flavonoids reduce the risk of developing dementia and have the potential to improve symptoms in individuals with Alzheimer’s disease [20,21]. The role of flavonoids and their potential impact as natural therapies in mental health and neurological disorders, such as Alzheimer’s and Parkinson’s diseases, is under investigation. Our knowledge of the roles and benefits of flavonoids on mental health and related diseases is limited and needs expansion, especially since flavonoids are abundantly available in plant-based foods and beverages [22,23].

Many studies in the literature describing associations between flavonoid intake and cognition have analyzed samples comprised primarily of White adults with average to above-average income and/or education [6,11,12,24]. As previously noted, the factors contributing to the disparities in lifetime risk of cognitive impairment among Black versus White adults warrant exploration. The Healthy Aging in Neighborhoods of Diversity across the Life Span (HANDLS) study provides the opportunity to investigate potential differences in flavonoid–cognition associations by race, as the sample is comprised of urban African American and White adults aged 30–64 from diverse socioeconomic backgrounds. Previous research documented significantly lower flavonoid intakes for African American HANDLS study participants compared to White study participants [25]. The objective of this study was to determine if associations exist between flavonoid intake and cognitive performance among a socioeconomically diverse sample, overall, and stratified by race, using the HANDLS study.

## 2. Methods

### 2.1. Study Sample

A total of 3720 adults aged 30–64 years were enrolled in the HANDLS study, all of whom resided in Baltimore City, Maryland, USA, in 13 specifically determined neighborhoods. Race was self-reported as African American or White. The baseline visit (visit 1) of this prospective cohort study was initiated in August 2004 and ended in March 2009. A detailed description of the study design and procedures is available online [26,27].

All participants were provided with a protocol booklet and watched a video that explained the study procedures before giving written informed consent [27]. The study protocol was approved by the Institutional Review Board of the National Institutes of Health. Data for this study, a secondary analysis, were derived from visit 1 and the third and fifth follow-up examinations (visit 3, 2009–2013, and visit 5, 2017–2020, respectively).

**Figure 1:**
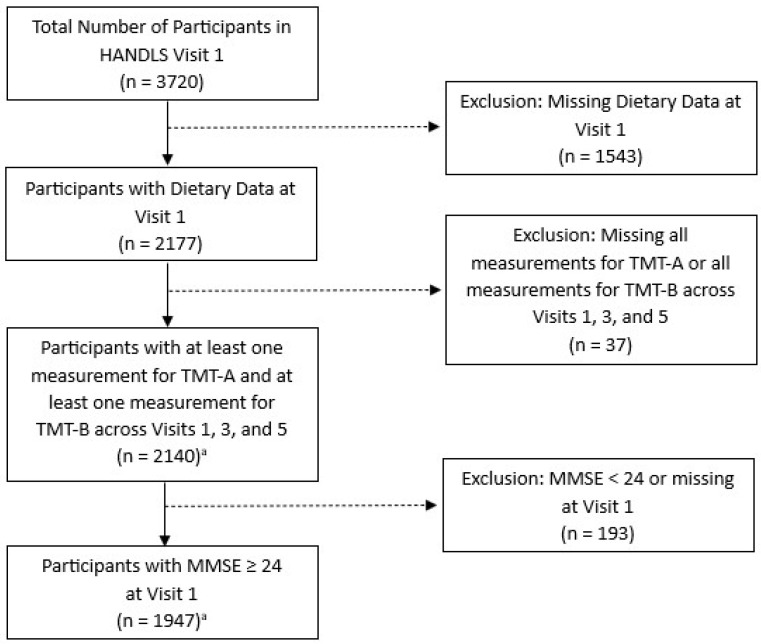
Flow diagram for study participation selection from Wave 1, HANDLS 2004–2009. Abbreviations: TMT, Trail Making Test, and MMSE, Mini-Mental State Exam. ^a^ The primary analytic sample consisted of the 1947 adults shown here. However, an additional analysis used the 2140 adults available prior to the exclusion for MMSE, while a second additional analysis used the 846 adults that remained after excluding 1101 individuals <50 years of age at visit 1.

The analytical sample consisted of 1947 participants with complete dietary data at visit 1 and at least one test result for both the Trail Making Test (TMT)-A and TMT-B, the response variables measuring cognitive performance, across visits 1, 3, and 5 (Figure 1). Sample participants were excluded if they had a score of <24 on the Mini-Mental State Exam (MMSE), a tool assessing the subset of cognitive status including attention, language, memory, orientation, and visuospatial proficiency [28]. A score of <24 indicates cognitive impairment [29].

### 2.2. Assessment of Flavonoid Intake

Intake of total flavonoids and five flavonoid classes was calculated from two 24-h dietary recalls collected in person by trained interviewers using the US Department of Agriculture (USDA) Automated Multiple Pass Method [30]. Participants were provided an illustrated food model booklet and measurement aids (ruler, measuring spoons, and cups) to assist them in recalling portion sizes of foods and beverages consumed. The USDA Food and Nutrient Database for Dietary Studies 3.0 was used to assign food codes with associated energy and nutrient profiles to all the foods and beverages reported in the recalls [31].

The USDA food codes provided the link between the dietary data and flavonoid composition data from the Database for Flavonoid Values for USDA Survey Food Codes 2007–2010 [32,33]. Mean dietary flavonoid intakes were estimated from foods and beverages only; no data were collected on baseline supplement intakes. For this study, 2-day mean intakes of energy, anthocyanidins, flavan-3-ols (inclusive of catechins, theaflavins, and thearubigins), flavanones, flavones, flavonols, and total flavonoids (summative total of the five previously listed classes plus isoflavones) were calculated.

### 2.3. Cognitive Measures

The TMT, a neuropsychological test, consists of two parts, A and B. It requires a variety of mental abilities for successful performance, including letter and number recognition, mental flexibility, visual scanning, and motor function [34]. TMT-A is generally regarded as measuring the attention, visual search, and motor skills domains that are associated with the non-dominant (right) cerebral hemisphere [35]. TMT-B is generally seen as a measure of the executive function, motor skills, speed of attention, and visual search domains, which are associated with the dominant (left) cerebral hemisphere [35].

Scores for both the TMT-A and TMT-B were obtained from participants at visits 1, 3, and 5. Prior to the start of both assessments, sample tests were performed to ensure the participant understood the task. In TMT-A, the participant drew a line to connect consecutive numbers, from 1 to 25. In TMT-B, the participant connected numbers and letters in an alternating progressive sequence, from a number to a letter, then a letter to a number, i.e., the sequence was from 1 to A, A to 2, 2 to B, B to 3, and so on. A stopwatch was used to record the time to complete each part, in seconds. The examiner started timing each part as soon as the instructions were completed and the participant was signaled to begin. Participants were allowed 5 min to complete each part of the TMT. The time to completion of TMT-A and TMT-B, in seconds, was recorded separately and used in the analyses.

### 2.4. Statistical Analyses

#### 2.4.1. Basic Model

Longitudinal mixed linear models were used to explore the associations between the response variables, TMT-A and TMT-B, collected over three time points (visits 1, 3, and 5) and visit 1 flavonoid intake. The aim of the modeling was to assess the associations between (1) flavonoid intake and TMT scores at visit 1 (cross-sectional) and (2) flavonoid intake at visit 1 and the change in TMT scores over time (longitudinal). The natural log (ln) of TMT-A and TMT-B was modeled, as both variables exhibited a strong right skew. A basic unadjusted model including only the variables needed to address the aims was fit, as well as three adjusted models that added covariates with the potential to affect test performance (e.g., age at baseline) to the basic model. The basic model included fixed effects for flavonoid intake at visit 1, time measured as years from visit 1, and the interaction between visit 1 flavonoid intake and time. It also included a random intercept with an unstructured covariance matrix to capture the correlation between repeated visits on a single subject. The fixed effects coefficient for visit 1 flavonoid intake captures the relationship between visit 1 flavonoid intake and visit 1 ln(TMT), addressing the first aim. In other words, it captures the effect of baseline flavonoid intake on ln(TMT) when time = 0 (i.e., at baseline visit 1) or the cross-sectional flavonoid intake–cognition relationships. The fixed effects coefficient for the interaction between visit 1 flavonoid intake and time captures the relationship between visit 1 flavonoid intake and the change in ln(TMT) over time, addressing the second aim (i.e., the longitudinal flavonoid intake–cognition relationships). The remaining three models used 15 covariates grouped as demographic, lifestyle, or clinical characteristics to adjust the basic statistical model.

#### 2.4.2. Demographic Model

The demographic model was adjusted to the basic model for the following covariates: baseline (visit 1) age in years; age-squared (to allow for a non-linear relationship between age and cognition); sex assigned at birth (male or female); self-reported race (African American or White); income; education; and literacy. Income was indexed by poverty below or above 125% of the 2004 US Health and Human Services Poverty Guidelines (125% equals a yearly income of USD 23,563 for a family of four in 2004) [36]. Education was captured in years. Literacy was measured via the Wide Range Achievement Test-3rd edition (WRAT3) reading score, which was calculated as the sum of total correctly pronounced letters and words [37].

#### 2.4.3. Lifestyle Model

In addition to the covariates included in the demographic model, the lifestyle model included current smoking status, current illicit drug use, and mean energy intake (kcal) based on two 24-h recalls. At the time of visit 1, illicit drugs were defined as marijuana, opiates, and cocaine. Cigarette smokers and users of drugs were categorized as current or never/former users [27].

#### 2.4.4. Clinical Model

In addition to the covariates included in the demographic model, the clinical model included diabetes, hypertension, high serum cholesterol, the Center for Epidemiologic Studies Depression Scale (CES-D) score, and body mass index (BMI). These covariates have been associated with cognitive performance [2,12,38,39]. Diabetes mellitus was based on three measures, namely, a fasting glucose level concentration of >126 mg/dL (7.0 mmol/L), self-reports, and/or taking medication for diabetes [27]. This variable was coded as a dichotomous variable (diabetic versus not diabetic); the pre-diabetic was grouped with the not diabetic group. Hypertension was defined as having an average of seated and standing systolic blood pressure > 140 mm Hg, an average of seated and standing diastolic blood pressure > 90 mm Hg [40], a history of blood pressure medication use, and/or a self-report of hypertension. Fasting venous blood specimens were collected from participants in the morning and analyzed by Quest Diagnostics, Inc. (Chantilly, VA, USA). The serum total cholesterol (mg/dL) was assessed using a spectrophotometer (Olympus 5400, Olympus, Melville, NY, USA). High serum cholesterol was defined as having a fasting blood level >200 mg/dL, self-reports, and/or taking medication to lower serum cholesterol. Depressive symptoms were measured by the 20-item CES-D, a symptom rating scale assessing self-reported depressed mood [41]. BMI (kg/m^2^) was calculated from measured weight and height. A calibrated Med-weigh model 2500 digital scale was used to measure weight. Height was measured with the HANDLS study participant’s heels and back against a stadiometer (Novel Products, Inc., Rockton, IL, USA).

#### 2.4.5. Missing Data

Several covariates had missing data: education (<1% of values), WRAT3 score (<1%), current smoking status (11%), current illicit drug use (30%), diabetes status (6%), hypertension status (16%), high cholesterol status (6%), CES-D (5%), and BMI (5%). Missing data for these variables were imputed using a discriminant function for the categorical variables and predictive mean matching for the continuous variables. Twenty imputed datasets were created. To avoid bias in estimates, all variables included in the analysis model were included in the imputation model [42]. Therefore, imputation models included ln(TMT-A), ln(TMT-B), race, time, baseline (visit 1) flavonoid intakes (total and class-specific), interactions between flavonoid intakes and time, age, age-squared, sex, poverty status, education, WRAT3 score, smoking status, illicit drug use, total energy intake, diabetes status, hypertension status, high cholesterol status, CES-D, and BMI.

#### 2.4.6. Model Detail

All statistical analyses were performed using SAS v 9.4. All models were fit for the entire analytic study sample and stratified by race. Separate models were used for total flavonoids and each flavonoid class (anthocyanidins, flavan-3-ols, flavanones, flavones, and flavonols). Two additional analyses were performed: (1) limiting the sample to adults ≥50 years of age and (2) including subjects that were initially excluded from the analytic sample based on visit 1 MMSE. Regression estimates were reported for a 10-unit increase in flavonoid intake. Statistical significance was set at α = 0.0083 after applying a Bonferroni adjustment of α = 0.05/6 to account for the 6 different flavonoid intake values being assessed.

## 3. Results

### 3.1. Study Sample Description

At visit 1, the analytical sample of n = 1947 adults had an average age of 48 years; 42% were male and 16% had diabetes (Table 1). The mean years of education was 12.3. Thirty percent of the sample had not completed high school. Among White adults (n = 823), 31% lived at <125% poverty, compared to 50% of African American adults (n = 1124). Approximately 56% of White participants had high serum cholesterol and 38% had hypertension, compared to 44% and 49% of African American participants, respectively. Moreover, approximately 47% of the overall sample were current cigarette smokers and 18% were current users of marijuana, opiates, and/or cocaine.

The intake of total flavonoids and all flavonoid classes was highly skewed. For total flavonoids, the mean intake was 253 mg with a standard error (SE) of 11.5 mg, whereas the median was 68.1 mg with an interquartile range (IQR) of 19.7–297.5 mg (Table 2). On a weight basis, flavan-3-ols accounted for approximately 85% of intake. The mean and median intake of flavan-3-ols was 214 mg (SE = 11.1 mg) and 14.7 mg (IQR = 3.3–248.1 mg), respectively, followed by flavonols (mean 18.1 mg, SE = 0.5 mg; and median 12.9 mg, (IQR = 6.2–23.7 mg)). The median score on the TMT-A was 31 s (IQR = 25–41 s) and the median score on the TMT-B was 87 s (IQR = 62–139 s).

### 3.2. Flavonoid-TMT A and B Associations

**Figure 2:**
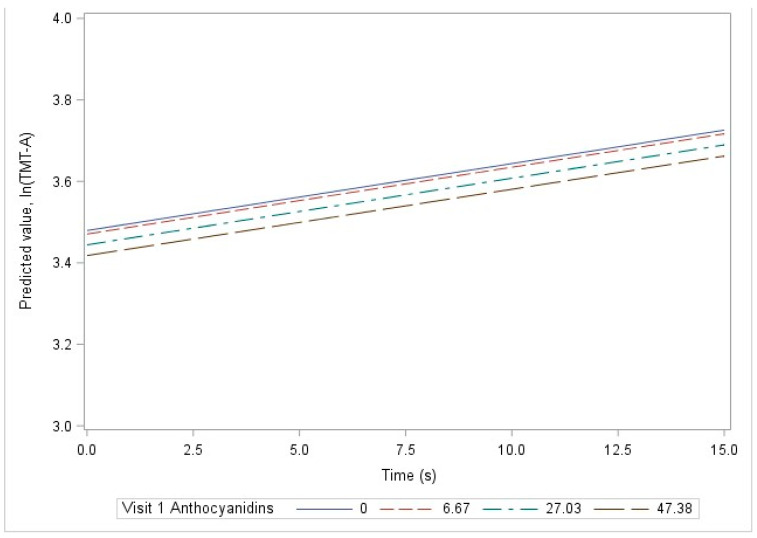
Linear prediction of ln(TMT-A), by time and visit 1 anthocyanidin levels, among all study subjects using the basic model, HANDLS 2004–2020.

Among all study subjects, the basic model demonstrated a significant association between visit 1 anthocyanidin intake and visit 1ln(TMT-A) scores. For a 10-unit (mg) increase in anthocyanidin intake, the ln(TMT-A) would be expected to decrease by 0.013 units at visit 1 (seconds; *p* = 0.006; Table 3). The association is depicted in Figure 2, where at time = 0 (i.e., visit 1), the predicted value for ln(TMT-B) is lower (i.e., predicted cognition is higher) for larger values of visit 1 anthocyanidin intake. This association was not significant after covariate adjustment in the demographic, lifestyle, or clinical models. There was no significant relationship between visit 1 flavonoid intake and change in ln(TMT-A) over time. An example of this lack of association over time is shown in Figure 2. The parallel lines indicate that differing levels of visit 1 anthocyanidin intake are not associated with differences in the rate of change in cognition over time. In addition, there were no significant associations between visit 1 flavonoid intake and either visit 1 ln(TMT-A) or change in ln(TMT-A) over time among White adults (Table S1) or African American adults (Table S2) when stratified by race.

There were several significant associations between visit 1 flavonoid intake and visit 1 ln(TMT-B) among all study subjects using the basic model. A significant decrease in ln(TMT-B) was observed for a 10-unit increase in intake of total flavonoids and all flavonoid classes except flavanones (Table 4). The significant associations included intake of total flavonoids ($\hat{\beta }$ = −0.001, *p* = 0.001), flavones ($\hat{\beta }$ = −0.887, *p* < 0.001), flavonols ($\hat{\beta }$ = −0.030, *p* < 0.001), flavan-3-ols ($\hat{\beta }$ = −0.001, *p* = 0.002), and anthocyanidins ($\hat{\beta }$ = −0.031, *p* < 0.001). However, there were no significant associations between visit 1 flavonoid intake and change in ln(TMT-B) over time. In addition, the significant associations did not persist after adjusting for covariates in the demographic, lifestyle, or clinical models.

Among White adults, significant associations between visit 1 flavonoid intake and visit 1 ln(TMT-B) exist for flavone intake ($\hat{\beta }$ = −0.745, *p*-value < 0.001) and anthocyanidin intake ($\hat{\beta }$ = −0.044, *p*-value < 0.001) in the basic model (Table 5). The only association to persist with further adjustment was that between anthocyanidin intake and ln(TMT-B) score and it was found in the demographic model only ($\hat{\;}$ = −0.022, *p*-value = 0.006). No significant associations were observed among African American adults (Table 6).

An additional analysis performed limited the study sample to adults ≥50 years of age (n = 846). No significant associations were seen between visit 1 flavonoid intake and visit 1 ln(TMT-A) or the change in ln(TMT-A) over time in any of the four models among all adults (Table S3), among White adults (Table S4), or among African American adults (Table S5). Using the basic model, visit 1 ln(TMT-B) exhibited expected decreases of 0.786 units for a 10-unit increase in visit 1 flavone intake (*p*-value = 0.003) and 0.037 units for a 10-unit increase in visit 1 anthocyanidin intake (*p*-value = 0.001) among all study subjects (Table S6). However, the associations did not persist after adjustment and no significant associations were observed between visit 1 flavonoid intake and change in ln(TMT-B) over time. The relationship between visit 1 anthocyanidin intake and visit 1 ln(TMT-B) using the basic model was also observed among White adults ($\hat{\beta }$ = −0.038, *p*-value = 0.001; Table S7) but not among African American adults (Table S8). No other significant associations were observed for ln(TMT-B) among White or African American adults aged 50 years and older.

In a second additional analysis (retaining individuals originally excluded because their visit 1 MMSE was <24), significant associations were observed with the basic model only between visit 1 flavonoid intake and visit 1 ln(TMT-A) among all adults for flavone intake ($\hat{\beta }$ = −0.309, *p* = 0.002), flavonols intake ($\hat{\beta }$ = −0.015, *p* = 0.002), and anthocyanidin intake ($\hat{\beta }$ = −0.017, *p* = 0.001) and among White adults for anthocyanidin intake ($\hat{\beta }$ = −0.017, *p* = 0.003) (Tables S9–S11). The findings between visit 1 flavonoid intake and visit 1 ln(TMT-B) were similar to those seen in the original study sample, with significant associations in the basic model only for total flavonoids and all flavonoid classes except flavanones among all adults (Table S12) and flavones and anthocyanidins among White adults (Tables S13 and S14).

## 4. Discussion

This study sought to determine if dietary flavonoid intake based on multiple 24-h dietary recalls was associated with cognitive function as assessed by the TMT-A and TMT-B scores. The associations evaluated included flavonoid intake and cognitive scores at visit 1 (cross-sectional) as well as flavonoid intake at visit 1 and cognitive scores over three study visits (longitudinal). To our knowledge, this is the first time that these relationships have been investigated in a sample of exclusively urban African American and White adults who present with multiple risk factors for diet-related disease, including cognitive impairment.

The basic model revealed several significant inverse associations between dietary intake of flavonoids at visit 1 and TMT-B scores at visit 1 for the overall sample and for White adults. In contrast, TMT-A scores were only inversely associated with anthocyanidin intake at visit 1 and only for the overall sample. Only the association between TMT-B and anthocyanidin intake at visit 1 among White adults was significant after covariate adjustment and only in the demographic model. Further adjustment of clinical and lifestyle characteristics mitigated the association. No other significant associations were found between flavonoid intake and TMT-A or TMT-B scores after covariate adjustments, either cross-sectionally or over time, for the overall sample, or among African American adults or White adults.

The null associations were not unanticipated. Results from research studies of flavonoid-cognitive associations have been inconsistent and even contradictory [43,44,45,46,47,48,49]. Those epidemiological and intervention studies that did find positive associations between the increased consumption of flavonoid-rich foods and cognitive function cite only weak effects [44,48]. For instance, Desideri and colleagues [45] and Mastroiacovo and colleagues [46] both noted slight improvements in TMT-A and TMT-B occurring with flavan-3-ol intakes of 520 or 993 mg, respectively, compared to 48 mg over 8 weeks while Brickman and colleagues [47] found an improvement only in TMT-B with flavan-3-ol intakes of 609 vs. 13 mg over 30 days. These findings suggest that cognitive change over time may not be detectable in both TMT-A and -B and that it may be dose-dependent. Other research has suggested such a dose-response relationship [50]. The total mean flavonoid intake of the HANDLS study participants was only 253 mg (median 68 mg, IQR 20–297 mg), which is well below the intakes cited in these previous studies showing positive associations. Additionally, the current recommendation for flavan-3-ols intake to reduce the risk of other chronic diseases, namely, cardiovascular disease and diabetes, is 400–600 mg/d [51] and the mean intake among HANDLS participants was only 214 mg (median 15 mg, IQR 3–248 mg). Furthermore, previous research reported that intakes of anthocyanidins and flavones were lower in the HANDLS study sample compared to the US population with similar demographic characteristics examined in What We Eat in America-National Health and Nutrition Examination Survey (WWEIA-NHANES) [32]. In HANDLS, it has been shown that diet quality as assessed by the dietary inflammatory index improved minimally over time [52], implying that the intake of flavonoids is also stable. It is plausible that flavonoid intakes in the HANDLS study were too low to detect any associations with cognitive measures.

The mechanisms by which flavonoids may improve cognitive function are not fully understood. The ability of flavonoids to improve cognition over short time frames may be mediated by peripheral vascular changes facilitating more efficient cerebral blood flow [43,44]. For example, anthocyanidins and their metabolites have neuroprotective actions, including decreasing neuroinflammation, preventing excitotoxicity, preventing aggregation of proteins, and activating pro-survival pathways while inhibiting pro-apoptotic pathways and improving axonal health [50]. They have been shown to enter the brain and their concentrations are correlated with cognitive performance [53,54], a possible explanation for the inverse associations seen between anthocyanidin intake and TMT-A and TMT-B in the basic models.

There is evidence that TMT performance declines in normal aging, with TMT-B performance declining more rapidly than TMT-A performance in older adults [55,56]. TMT-B is considered a measure of fluid intelligence [57]. Many fluid cognitive abilities, especially psychomotor ability and processing speed, peak in the third decade of life and then decline at an estimated rate of −0.02 standard deviations per year [58]. Evidence suggests that the effects of flavonoids on cognition are mediated by age and are dose-dependent [50]. The age of the sample at visit 1 ranged from 30 to 64 years with a mean follow-up of 12.1 years among those subjects that completed visit 5, suggesting many participants were at or close to their cognitive peak at baseline. Consequently, it is expected that any flavonoid–cognition associations observed would only concern cognitive measures of more complex abilities, such as those reflected in the TMT-B [50]. The observed minimally adjusted associations between flavonoid intake and TMT-B might reflect the potential for improvements in the complex abilities that comprise the executive function cognitive domain.

It is well-established that healthy dietary patterns are associated with more favorable cognitive outcomes [58]. Overall, diet quality was low in HANDLS. The mean score for diet quality evaluated by the Healthy Eating Index-2010 score of the HANDLS study participants at visit 1 was 42.6 out of a possible 100 points with a range of 42–49 [52], compared to a range of mean scores between 57.4 and 61.6 for US individuals of similar ages [59]. In addition, and perhaps at least as important, is the impact of other lifestyle behaviors on cognitive outcomes that could attenuate any dietary influences on the brain [56]. The HANDLS study sample has an extremely high prevalence of unhealthy lifestyle behaviors, as evidenced by about 47% of the sample being classified as current smokers, as compared to about 21% nationally in 2004 [60]. Smoking is widely associated with cognitive decline [61,62,63]. The prevalence of chronic diseases among HANDLS study participants is also high, often higher than the US national population. For example, at the baseline visit of the HANDLS study, the prevalence of obesity was 42.1% [64]; diabetes, 16.5% [65]; and hypertension, 45.2% [66]. In comparison, national estimates among adults for a similar time frame were 32.2% for obesity [67], 10.3% for diabetes [68], and 29.3% for hypertension [69]. The benefits of flavonoids may be muted not only by their low intake but also by the high prevalence of other lifestyle behaviors and health conditions that accelerate cognitive decline.

This study has a few limitations. First, the 24-h dietary recalls were self-reported and could have been affected by memory, aspects of cognitive reserve like education and income, and social desirability bias [70,71]. However, the dietary data collection method, the automated multiple-pass method, has been validated using the doubly labeled water technique and has been shown to reduce bias in the collection of energy intakes [72]. Second, nutritional supplement intake was not obtained at visit 1 but could impact flavonoid intake and thus flavonoid–cognition associations. Lastly, due to data availability, only one cognitive measure was used in the analyses.

This study has several strengths. First, the flavonoid database is comprehensive, containing values for total flavonoids in six classes for over 7000 foods and beverages [33]. Second, the stratified design of the HANDLS study with the relatively large number of African American participants allowed the investigation of associations by race [26]. Moreover, with the added dimensions of income (low to moderate) and residence (exclusively urban), the HANDLS study participants represent a socioeconomically disadvantaged group understudied in the literature. Third, the availability of TMT-A and B over three time points allowed longitudinal, as well as cross-sectional, analyses. Finally, a variety of confounders not related to diet were accounted for in the analyses.

## 5. Conclusions

Minimally adjusted analyses suggest beneficial associations between dietary intake of flavonoids and cognitive scores, as assessed via the TMT-A and TMT-B scores. However, accounting for confounders attenuated these relationships. The network of brain regions used to successfully perform the TMT-A and TMT-B and the potential impact of flavonoids on these regions has not yet been studied in sufficient detail and warrants further investigation. In order to advance the field and make conclusive statements based on evidence, it is important to continue the study of the relationship between flavonoid intake and cognition in samples with a wide range of flavonoid intake, especially individuals with intakes with high intakes.

## Figures and Tables

**Table 1:** Description of visit 1 characteristics, overall analytical sample, and race, HANDLS 2004–2009.

Characteristic	All Adults (n = 1947)	White Adults (n = 823)	African American Adults (n = 1124)
	$\bar{X}\pm SE$	$\bar{X}\pm SE$	$\bar{X}\pm SE$
Energy Intake (kcal/d)	2015 ± 22	2036 ± 32	1999 ± 30
Age, years	48.06 ± 0.21	48.28 ± 0.33	47.90 ± 0.28
WRAT3 score	43.09 ± 0.16	45.89 ± 0.23	41.05 ± 0.21
CES-D	14.58 ± 0.25	14.80 ± 0.40	14.41 ± 0.32
Body Mass Index	29.98 ± 0.18	30.05 ± 0.26	29.93 ± 0.23
Education (yrs)	12.31 ± 0.06	12.56 ± 0.10	12.13 ± 0.07
	**% ± SE**	**% ± SE**	**% ± SE**
Male Sex	41.60 ± 1.12	40.58 ± 1.71	42.35 ± 1.47
Poverty < 125% ^a^	42.01 ± 1.12	31.11 ± 1.61	50.00 ± 1.49
High Cholesterol	49.25 ± 1.18	56.37 ± 1.81	44.03 ± 1.56
Diabetes	15.83 ± 0.83	15.80 ± 1.29	15.85 ± 1.10
Hypertension	44.58 ± 1.14	38.13 ± 1.72	49.31 ± 1.50
Current smoker	47.24 ± 1.16	43.78 ± 1.80	49.78 ± 1.54
Current drug user ^b^	17.84 ± 0.89	12.29 ± 1.18	21.90 ± 1.27

Abbreviations: SE, standard error; WRAT3, Wide Range Achievement Test-3rd ed; CES-D, Center of Epidemiological Studies-Depression; kcal/d, kilocalories/day; yrs, years. ^a^ Poverty < 125% of 2004 DHHS levels [36]. ^b^ Drug use is defined as illicit drugs, specifically, marijuana, cocaine, and opiates.

**Table 2:** Visit 1 flavonoid intake and cognitive function, HANDLS 2004–2009.

Characteristic	$\bar{X}\pm SE$	Median	IQR (Q1–Q3)
Total	253.31 ± 11.54	68.11	(19.66–297.47)
Flavonols	18.14 ± 0.46	12.85	(6.18–23.69)
Flavones	0.62 ± 0.02	0.32	(0.11–0.76)
Flavanones	12.44 ± 0.62	0.38	(0.00–11.82)
Flavan-3-ols	214.43 ± 11.13	14.73	(3.30–248.11)
Anthocyanins	6.61 ± 0.45	0.36	(0.00–3.39)
TMT-A	37.88 ± 1.06	31.00	(25.00–41.00)
TMT-B	144.58 ± 3.53	87.00	(62.00–139.00)

Abbreviations: SE, standard error; IQR, interquartile range; Q, quartile; mg, milligrams; sec, seconds; TMT, Trails Making Test.

**Table 3:** Association ^a^ between visit 1 flavonoid intake and ln(TMT-A) for all study participants, HANDLS 2004–2020.

	Basic Model ^b^		Demographic Model ^c^		Lifestyle Model ^d^		Clinical Model ^e^	
	$\hat{\beta }\;\pm$ SE	*p*-Value	$\hat{\beta }\;\pm$ SE	*p*-Value	$\hat{\beta }\;\pm$ SE	*p*-Value	$\hat{\beta }\pm$ SE	*p*-Value
*Flavonoid Main Effect: Association between visit 1 flavonoid intake and visit 1 ln(TMT-A)*								
Total Flavonoids	−0.00034 ± 0.00019	0.065	0.00011 ± 0.00017	0.523	0.00014 ± 0.00017	0.401	0.00005 ± 0.00017	0.759
Flavones	−0.20026 ± 0.09613	0.037	0.08503 ± 0.08817	0.335	0.13137 ± 0.08862	0.138	0.08991 ± 0.08762	0.305
Flavonols	−0.01021 ± 0.00468	0.029	0.00131 ± 0.00426	0.759	0.00491 ± 0.00433	0.256	0.00067 ± 0.00424	0.874
Flavonones	−0.00209 ± 0.00350	0.549	−0.00461 ± 0.00316	0.144	−0.00308 ± 0.00318	0.332	−0.00399 ± 0.00314	0.205
Flavan-3-ols	−0.00032 ± 0.00019	0.096	0.00013 ± 0.00018	0.453	0.00015 ± 0.00018	0.381	0.00007 ± 0.00017	0.697
Anthocyanidins	−0.01301 ± 0.00473	0.006	−0.00395 ± 0.00432	0.361	−0.00199 ± 0.00434	0.647	−0.00281 ± 0.00431	0.514
Flavonoid*Time Interaction: Association between visit 1 flavonoid intake and change in ln(TMT-A) over time								
Total Flavonoids	−0.00001 ± 0.00002	0.823	−0.00001 ± 0.00002	0.618	−0.00001 ± 0.00002	0.634	−0.00001 ± 0.00002	0.631
Flavones	−0.02099 ± 0.01207	0.082	−0.02334 ± 0.01195	0.051	−0.02449 ± 0.01196	0.041	−0.02318 ± 0.01195	0.052
Flavonols	0.00003 ± 0.00059	0.964	−0.00012 ± 0.00059	0.838	−0.00010 ± 0.00059	0.863	−0.00015 ± 0.00059	0.798
Flavonones	−0.00017 ± 0.00043	0.690	−0.00027 ± 0.00043	0.526	−0.00028 ± 0.00043	0.506	−0.00024 ± 0.00043	0.569
Flavan-3-ols	0.00000 ± 0.00002	0.864	−0.00001 ± 0.00002	0.673	−0.00001 ± 0.00002	0.688	−0.00001 ± 0.00002	0.682
Anthocyanidins	−0.00003 ± 0.00050	0.953	−0.00018 ± 0.00049	0.719	−0.00018 ± 0.00049	0.717	−0.00014 ± 0.00049	0.770

Abbreviations: TMT, Trail Making Test; SE, standard error. ^a^ Associations are reported for a 10-unit increment in visit 1 flavonoid intake. ^b^ Basic model includes fixed effects for visit 1 flavonoid intake, time, and visit 1 flavonoid intake*time. ^c^ Demographic model is the basic model adjusted for visit 1 age in years, age-squared, sex, race, poverty status, education in years, and Wide Range Achievement Test (WRAT) scores. ^d^ Lifestyle model is the demographic model adjusted for current smoking status, current drug use, and total energy intake at visit 1. ^e^ Clinical model is the demographic model adjusted for diabetes, hypertension, high cholesterol, the Center for Epidemiologic Studies Depression Scale (CES-D), and body mass index (BMI).

**Table 4:** Association ^a^ between visit 1 flavonoid intake and ln(TMT-B) for all study participants, HANDLS 2004–2020.

	Basic Model ^b^		Demographic Model ^c^		Lifestyle Model ^d^		Clinical Model ^e^	
	$\hat{\beta }\;\pm SE$	*p*-Value	$\hat{\beta }\;\pm SE$	*p*-Value	$\hat{\beta }\;\pm SE$	*p*-Value	$\hat{\beta }\;\pm SE$	*p*-Value
*Flavonoid Main Effect: Association between visit 1 flavonoid intake and visit 1 ln(TMT-B)*								
Total Flavonoids	−0.00105 ± 0.00031	0.001	−0.00015 ± 0.00027	0.586	−0.00011 ± 0.00027	0.684	−0.00022 ± 0.00026	0.410
Flavones	−0.88659 ± 0.16112	<0.001	−0.15912 ± 0.13936	0.254	−0.10337 ± 0.14033	0.461	−0.13482 ± 0.13754	0.327
Flavonols	−0.03028 ± 0.00787	<0.001	−0.00363 ± 0.00673	0.590	0.00078 ± 0.00685	0.910	−0.00422 ± 0.00665	0.526
Flavonones	0.00860 ± 0.00589	0.144	0.00591 ± 0.00499	0.237	0.00813 ± 0.00502	0.106	0.00738 ± 0.00493	0.134
Flavan-3-ols	−0.00103 ± 0.00033	0.002	−0.00016 ± 0.00028	0.569	−0.00014 ± 0.00028	0.621	−0.00025 ± 0.00027	0.372
Anthocyanidins	−0.03067 ± 0.00799	<0.001	−0.00381 ± 0.00686	0.578	−0.00120 ± 0.00689	0.862	−0.00115 ± 0.00678	0.865
Flavonoid*Time Interaction: Association between visit 1 flavonoid intake and change in ln(TMT-B) over time								
Total Flavonoids	0.00007 ± 0.00003	0.033	0.00006 ± 0.00003	0.070	0.00006 ± 0.00003	0.067	0.00005 ± 0.00003	0.091
Flavones	0.01514 ± 0.01644	0.358	0.01254 ± 0.01630	0.442	0.01169 ± 0.01633	0.474	0.01189 ± 0.01629	0.466
Flavonols	0.00177 ± 0.00082	0.031	0.00152 ± 0.00081	0.059	0.00154 ± 0.00081	0.057	0.00131 ± 0.00081	0.105
Flavonones	−0.00038 ± 0.00059	0.521	−0.00052 ± 0.00059	0.370	−0.00054 ± 0.00059	0.360	−0.00055 ± 0.00059	0.345
Flavan-3-ols	0.00007 ± 0.00003	0.033	0.00006 ± 0.00003	0.067	0.00006 ± 0.00003	0.064	0.00006 ± 0.00003	0.085
Anthocyanidins	0.00044 ± 0.00068	0.520	0.00027 ± 0.00067	0.684	0.00026 ± 0.00067	0.697	0.00025 ± 0.00067	0.714

Abbreviations: TMT, Trail Making Test; SE, standard error. ^a^ Associations are reported for a 10-unit increment in visit 1 flavonoid intake. ^b^ Basic model includes fixed effects for visit 1 flavonoid intake, time, and visit 1 flavonoid intake*time. ^c^ Demographic model is the basic model adjusted for visit 1 age in years, age-squared, sex, race, poverty status, education in years, and Wide Range Achievement Test (WRAT) scores. ^d^ Lifestyle model is the demographic model adjusted for current smoking status, current drug use, and total energy intake at visit 1. ^e^ Clinical model is the demographic model adjusted for diabetes, hypertension, high cholesterol, the Center for Epidemiologic Studies Depression Scale (CES-D), and body mass index (BMI).

**Table 5:** Association ^a^ between visit 1 flavonoid intake and ln(TMT-B) for White study participants, HANDLS 2004–2020.

	Basic Model ^b^		Demographic Model ^c^		Lifestyle Model ^d^		Clinical Model ^e^	
	$\hat{\beta }\;\pm SE$	*p*-Value	$\hat{\beta }\;\pm SE$	*p*-Value	$\hat{\beta }\;\pm SE$	*p*-Value	$\hat{\beta }\;\pm SE$	*p*-Value
*Flavonoid Main Effect: Association between visit 1 flavonoid intake and visit 1 ln(TMT-B)*								
Total Flavonoids	−0.00027 ± 0.00030	0.371	−0.00018 ± 0.00026	0.505	−0.00016 ± 0.00026	0.539	−0.00024 ± 0.00026	0.364
Flavones	−0.74494 ± 0.17815	<0.001	−0.34641 ± 0.15731	0.028	−0.31370 ± 0.15890	0.048	−0.33389 ± 0.15590	0.032
Flavonols	−0.01483 ± 0.00817	0.070	−0.00545 ± 0.00705	0.439	−0.00389 ± 0.00713	0.585	−0.00635 ± 0.00700	0.364
Flavonones	−0.01065 ± 0.00967	0.271	−0.00173 ± 0.00845	0.838	−0.00008 ± 0.00850	0.992	0.00119 ± 0.00839	0.887
Flavan-3-ols	−0.00019 ± 0.00032	0.538	−0.00015 ± 0.00027	0.588	−0.00014 ± 0.00027	0.610	−0.00022 ± 0.00027	0.420
Anthocyanidins	−0.04363 ± 0.00896	<0.001	−0.02208 ± 0.00802	0.006	−0.02080 ± 0.00806	0.010	−0.02031 ± 0.00797	0.011
Flavonoid*Time Interaction: Association between visit 1 flavonoid intake and change in ln(TMT-B) over time								
Total Flavonoids	0.00005 ± 0.00003	0.118	0.00004 ± 0.00003	0.211	0.00004 ± 0.00003	0.204	0.00004 ± 0.00003	0.221
Flavones	0.02941 ± 0.01983	0.138	0.02882 ± 0.01967	0.143	0.02839 ± 0.01973	0.150	0.03199 ± 0.01963	0.103
Flavonols	0.00161 ± 0.00087	0.063	0.00133 ± 0.00086	0.120	0.00135 ± 0.00086	0.117	0.00125 ± 0.00086	0.145
Flavonones	−0.00033 ± 0.00117	0.778	−0.00042 ± 0.00115	0.713	−0.00046 ± 0.00116	0.693	−0.00050 ± 0.00115	0.664
Flavan-3-ols	0.00005 ± 0.00003	0.133	0.00004 ± 0.00003	0.230	0.00004 ± 0.00003	0.221	0.00004 ± 0.00003	0.237
Anthocyanidins	0.00112 ± 0.00081	0.169	0.00091 ± 0.00080	0.259	0.00088 ± 0.00081	0.274	0.00085 ± 0.00080	0.287

Abbreviations: TMT, Trail Making Test; SE, standard error. ^a^ Associations are reported for a 10-unit increment in visit 1 flavonoid intake. ^b^ Basic model includes fixed effects for visit 1 flavonoid intake, time, and visit 1 flavonoid intake*time. ^c^ Demographic model is the basic model adjusted for visit 1 age in years, age-squared, sex, race, poverty status, education in years, and Wide Range Achievement Test (WRAT) scores. ^d^ Lifestyle model is the demographic model adjusted for current smoking status, current drug use, and total energy intake at visit 1. ^e^ Clinical model is the demographic model adjusted for diabetes, hypertension, high cholesterol, the Center for Epidemiologic Studies Depression Scale (CES-D), and body mass index (BMI).

**Table 6:** Association ^a^ between visit 1 flavonoid intake and ln(TMT-B) for African American study participants, HANDLS 2004–2020.

	Basic Model ^b^		Demographic Model ^c^		Lifestyle Model ^d^		Clinical Model ^e^	
	$\hat{\beta }\;\pm SE$	*p*-Value	$\hat{\beta }\;\pm SE$	*p*-Value	$\hat{\beta }\;\pm SE$	*p*-Value	$\hat{\beta }\;\pm SE$	*p*-Value
*Flavonoid Main Effect: Association between visit 1 flavonoid intake and visit 1 ln(TMT-B)*								
Total Flavonoids	−0.00044 ± 0.00078	0.572	0.00028 ± 0.00069	0.689	0.00041 ± 0.00069	0.552	0.00037 ± 0.00068	0.584
Flavones	−0.30104 ± 0.27141	0.268	0.12748 ± 0.24401	0.601	0.21299 ± 0.24538	0.385	0.15898 ± 0.24035	0.508
Flavonols	−0.00647 ± 0.01531	0.673	0.00451 ± 0.01380	0.744	0.01712 ± 0.01434	0.233	0.00721 ± 0.01360	0.596
Flavonones	0.00499 ± 0.00705	0.479	0.00868 ± 0.00629	0.168	0.01140 ± 0.00634	0.072	0.00915 ± 0.00620	0.140
Flavan-3-ols	−0.00057 ± 0.00081	0.481	0.00008 ± 0.00072	0.914	0.00014 ± 0.00072	0.847	0.00015 ± 0.00071	0.828
Anthocyanidins	0.01909 ± 0.01283	0.137	0.02348 ± 0.01144	0.040	0.02879 ± 0.01153	0.013	0.02559 ± 0.01127	0.023
Flavonoid*Time Interaction: Association between visit 1 flavonoid intake and change in ln(TMT-B) over time								
Total Flavonoids	0.00008 ± 0.00008	0.293	0.00008 ± 0.00008	0.329	0.00008 ± 0.00008	0.318	0.00006 ± 0.00008	0.444
Flavones	−0.01426 ± 0.02592	0.582	−0.01687 ± 0.02578	0.513	−0.01925 ± 0.02584	0.456	−0.02176 ± 0.02583	0.399
Flavonols	0.00108 ± 0.00159	0.497	0.00110 ± 0.00158	0.486	0.00113 ± 0.00158	0.475	0.00056 ± 0.00158	0.723
Flavonones	−0.00025 ± 0.00071	0.727	−0.00040 ± 0.00071	0.577	−0.00041 ± 0.00071	0.563	−0.00041 ± 0.00071	0.562
Flavan-3-ols	0.00010 ± 0.00008	0.241	0.00009 ± 0.00008	0.264	0.00009 ± 0.00008	0.255	0.00008 ± 0.00008	0.359
Anthocyanidins	−0.00108 ± 0.00109	0.321	−0.00106 ± 0.00108	0.325	−0.00108 ± 0.00108	0.317	−0.00109 ± 0.00108	0.313

Abbreviations: TMT, Trail Making Test; SE, standard error. ^a^ Associations are reported for a 10-unit increment in visit 1 flavonoid intake. ^b^ Basic model includes fixed effects for visit 1 flavonoid intake, time, and visit 1 flavonoid intake*time. ^c^ Demographic model is the basic model adjusted for visit 1 age in years, age-squared, sex, race, poverty status, education in years, and Wide Range Achievement Test (WRAT) scores. ^d^ Lifestyle model is the demographic model adjusted for current smoking status, current drug use, and total energy intake at visit 1. ^e^ Clinical model is the demographic model adjusted for diabetes, hypertension, high cholesterol, the Center for Epidemiologic Studies Depression Scale (CES-D), and body mass index (BMI).

## Data Availability

Data are available upon request to researchers with valid proposals who agree to the confidentiality agreement as required by our Institutional Review Board. We publicize our policies on our website https://handls.nih.gov (accessed 12 February 2024). Requests for data access may be sent to Alan Zonderman (co-author) or the study manager, Jennifer Norbeck, at norbeckje@mail.nih.gov.

## Supplementary Materials

The following supporting information can be downloaded at https://www.mdpi.com/article/10.3390/nu16091360/s1. Table S1: Association between visit 1 flavonoid intake and ln(TMT-A) for White study participants, HANDLS 2004–2020; Table S2: Association between visit 1 flavonoid intake and ln(TMT-A) for African American study participants, HANDLS 2004–2020; Table S3: Association between visit 1 flavonoid intake and ln(TMT-A) for all study participants 50+ years, HANDLS 2004–2020; Table S4: Association between visit 1 flavonoid intake and ln(TMT-A) for White study participants 50+ years, HANDLS 2004–2020; Table S5: Association between visit 1 flavonoid intake and ln(TMT-A) for African American study participants 50+ years, HANDLS 2004–2020; Table S6: Association between visit 1 flavonoid intake and ln(TMT-B) for all study participants 50+ years, HANDLS 2004–2020; TableS7: Association between visit 1 flavonoid intake and ln(TMT-B) for White study participants 50+ years, HANDLS 2004–2020; Table S8: Association between visit 1 flavonoid intake and ln(TMT-B) for African American study participants 50+ years, HANDLS 2004–2020; Table S9: Association between visit 1 flavonoid intake and ln(TMT-A) for all study participants without MMSE exclusion, HANDLS 2004–2020; Table S10: Association between visit 1 flavonoid intake and ln(TMT-A) for White study participants without MMSE exclusion, HANDLS 2004–2020; Table S11: Association between visit 1 flavonoid intake and ln(TMT-A) for African American study participants without MMSE exclusion, HANDLS 2004–2020; Table S12: Association between visit 1 flavonoid intake and ln(TMT-B) for all study participants without MMSE exclusion, HANDLS 2004–2020; TableS13: Association between visit 1 flavonoid intake and ln(TMT-B) for White study participants without MMSE exclusion, HANDLS 2004–2020; Table S14: Association between visit 1 flavonoid intake and ln(TMT-B) for African American study participants without MMSE exclusion, HANDLS 2004–2020.
