# Supplementary material for: Association between Flavonoid Intake and Cognitive Executive Function among African American and White Adults in the Healthy Aging in Neighborhoods of Diversity across the Life Span (HANDLS) Study
Source: Nutrients. 2024 Apr 30;16(9):1360. doi: 10.3390/nu16091360 (PMC11085386; doi:10.3390/nu16091360)
Supplement: Supplementary file 1 [file nutrients-16-01360-s001.zip › nutrients-2907365-supplementary.pdf]

Table S1. Association[a] between visit 1 flavonoid intake and ln(TMT-A) for White study participants, HANDLS 2004-2020.

	Basic Model[b]				Demographic Model[c]				Lifestyle Model[d]				Clinical Model[e]			
	$\hat{\beta}$	±	SE	p-value	$\hat{\beta}$	±	SE	p-value	$\hat{\beta}$	±	SE	p-value	$\hat{\beta}$	±	SE	p-value
Flavonoid Main Effect: Association between visit 1 flavonoid intake and visit 1 ln(TMT-A)																
Total Flavonoids	0.00000	±	0.00019	0.985	0.00009	±	0.00018	0.621	0.00012	±	0.00018	0.520	0.00003	±	0.00018	0.860
Flavones	-0.12931	±	0.11346	0.255	0.01560	±	0.10762	0.885	0.06186	±	0.10835	0.568	0.01463	±	0.10702	0.891
Flavonols	-0.00341	±	0.00515	0.508	0.00075	±	0.00480	0.876	0.00300	±	0.00484	0.535	-0.00020	±	0.00479	0.966
Flavonones	-0.00463	±	0.00614	0.450	-0.00362	±	0.00578	0.531	-0.00188	±	0.00580	0.745	-0.00330	±	0.00576	0.567
Flavan-3-ols	0.00002	±	0.00020	0.901	0.00011	±	0.00019	0.566	0.00013	±	0.00019	0.491	0.00005	±	0.00019	0.807
Anthocyanidins	-0.01383	±	0.00568	0.015	-0.00827	±	0.00545	0.129	-0.00692	±	0.00547	0.206	-0.00785	±	0.00544	0.149
*Flavonoid*Time Interaction: Association between visit 1 flavonoid intake and change in ln(TMT-A) over time*																
Total Flavonoids	-0.00001	±	0.00002	0.618	-0.00002	±	0.00002	0.531	-0.00002	±	0.00002	0.529	-0.00001	±	0.00002	0.702
Flavones	-0.03148	±	0.01504	0.037	-0.03107	±	0.01496	0.038	-0.03172	±	0.01497	0.034	-0.02892	±	0.01493	0.053
Flavonols	-0.00045	±	0.00065	0.488	-0.00052	±	0.00065	0.421	-0.00053	±	0.00065	0.417	-0.00037	±	0.00065	0.563
Flavonones	0.00051	±	0.00087	0.562	0.00049	±	0.00087	0.573	0.00043	±	0.00087	0.618	0.00047	±	0.00086	0.586
Flavan-3-ols	-0.00001	±	0.00003	0.625	-0.00002	±	0.00003	0.545	-0.00002	±	0.00003	0.545	-0.00001	±	0.00003	0.713
Anthocyanidins	0.00002	±	0.00061	0.976	-0.00017	±	0.00061	0.781	-0.00018	±	0.00061	0.763	-0.00012	±	0.00061	0.841

Abbreviations: TMT, Trail Making Test; SE, Standard Error.

[a]Associations are reported for a 10-unit increment in visit 1 flavonoid intake.

[b]Basic Model includes fixed effects for visit 1 flavonoid inake, time, and visit 1 flavonoid intake*time.

[c]Demographic Model is the Basic Model adjusted for visit 1 age in years, age-squared, sex, race, poverty status, education in years, and Wide Range Achievement Test (WRAT) scores.

[d]Lifestyle Model is the Demographic Model adjusted for current smoking status, current drug use, and total energy intake at visit 1.

[e]Clinical Model is the Demographic Model adjusted for diabetes, hypertension, high cholesterol, the Center for Epidemiologic Studies Depression Scale (CES-D), and body mass index (BMI).

Table S2. Association[a] between visit 1 flavonoid intake and ln(TMT-A) for African American study participants, HANDLS 2004-2020.

	Basic Model[b]				Demographic Model[c]				Lifestyle Model[d]				Clinical Model[e]			
	$\hat{\beta}$	±	SE	p-value	$\hat{\beta}$	±	SE	p-value	$\hat{\beta}$	±	SE	p-value	$\hat{\beta}$	±	SE	p-value
Flavonoid Main Effect: Association between visit 1 flavonoid intake and visit 1 ln(TMT-A)																
Total Flavonoids	-0.00016	±	0.00046	0.733	0.00009	±	0.00042	0.837	0.00017	±	0.00042	0.686	0.00012	±	0.00042	0.783
Flavones	0.05521	±	0.16015	0.730	0.18620	±	0.14808	0.209	0.23777	±	0.14885	0.110	0.18686	±	0.14720	0.204
Flavonols	-0.00103	±	0.00908	0.910	0.00039	±	0.00843	0.963	0.00809	±	0.00873	0.354	0.00153	±	0.00838	0.855
Flavonones	-0.00577	±	0.00416	0.166	-0.00435	±	0.00383	0.256	-0.00306	±	0.00386	0.428	-0.00415	±	0.00381	0.276
Flavan-3-ols	-0.00009	±	0.00048	0.843	0.00013	±	0.00044	0.765	0.00017	±	0.00044	0.694	0.00016	±	0.00044	0.721
Anthocyanidins	0.00083	±	0.00760	0.913	0.00159	±	0.00698	0.820	0.00455	±	0.00704	0.518	0.00215	±	0.00695	0.757
*Flavonoid*Time Interaction: Association between visit 1 flavonoid intake and change in ln(TMT-A) over time*																
Total Flavonoids	0.00004	±	0.00006	0.498	0.00003	±	0.00006	0.612	0.00003	±	0.00006	0.588	0.00002	±	0.00006	0.744
Flavones	-0.00980	±	0.01879	0.602	-0.01346	±	0.01861	0.469	-0.01555	±	0.01863	0.404	-0.01533	±	0.01864	0.411
Flavonols	0.00126	±	0.00114	0.270	0.00118	±	0.00113	0.294	0.00121	±	0.00113	0.284	0.00090	±	0.00113	0.424
Flavonones	-0.00040	±	0.00051	0.435	-0.00055	±	0.00051	0.276	-0.00056	±	0.00051	0.269	-0.00053	±	0.00051	0.296
Flavan-3-ols	0.00005	±	0.00006	0.443	0.00004	±	0.00006	0.528	0.00004	±	0.00006	0.506	0.00003	±	0.00006	0.661
Anthocyanidins	-0.00006	±	0.00079	0.940	-0.00004	±	0.00078	0.961	-0.00004	±	0.00078	0.958	-0.00002	±	0.00078	0.981

Abbreviations: TMT, Trail Making Test; SE, Standard Error.

[a]Associations are reported for a 10-unit increment in visit 1 flavonoid intake.

[b]Basic Model includes fixed effects for visit 1 flavonoid inake, time, and visit 1 flavonoid intake*time.

[c]Demographic Model is the Basic Model adjusted for visit 1 age in years, age-squared, sex, race, poverty status, education in years, and Wide Range Achievement Test (WRAT) scores.

[d]Lifestyle Model is the Demographic Model adjusted for current smoking status, current drug use, and total energy intake at visit 1.

[e]Clinical Model is the Demographic Model adjusted for diabetes, hypertension, high cholesterol, the Center for Epidemiologic Studies Depression Scale (CES-D), and body mass index (BMI).

Table S3. Association[a] between visit 1 flavonoid intake and ln(TMT-A) for all study participants 50+ years, HANDLS 2004-2020.

	Basic Model[b]				Demographic Model[c]				Lifestyle Model[d]				Clinical Model[e]			
	$\widehat{\beta}$	±	SE	p-value	$\widehat{\beta}$	±	SE	p-value	$\widehat{\beta}$	±	SE	p-value	$\widehat{\beta}$	±	SE	p-value
Flavonoid Main Effect: Association between visit 1 flavonoid intake and visit 1 ln(TMT-A)																
Total Flavonoids	-0.00087	±	0.00034	0.010	-0.00022	±	0.00031	0.488	-0.00015	±	0.00031	0.627	-0.00027	±	0.00031	0.385
Flavones	-0.24376	±	0.15976	0.127	0.10282	±	0.15042	0.494	0.17461	±	0.15118	0.248	0.11087	±	0.14926	0.458
Flavonols	-0.01972	±	0.00790	0.013	-0.00456	±	0.00737	0.536	0.00091	±	0.00751	0.903	-0.00419	±	0.00730	0.567
Flavonones	-0.00381	±	0.00553	0.491	-0.00345	±	0.00511	0.499	-0.00103	±	0.00516	0.843	-0.00305	±	0.00506	0.547
Flavan-3-ols	-0.00084	±	0.00035	0.017	-0.00022	±	0.00033	0.506	-0.00018	±	0.00032	0.588	-0.00028	±	0.00032	0.389
Anthocyanidins	-0.01581	±	0.00627	0.012	-0.00224	±	0.00586	0.702	0.00058	±	0.00590	0.922	-0.00132	±	0.00582	0.820
*Flavonoid*Time Interaction: Association between visit 1 flavonoid intake and change in ln(TMT-A) over time*																
Total Flavonoids	0.00006	±	0.00005	0.211	0.00005	±	0.00005	0.251	0.00006	±	0.00005	0.206	0.00005	±	0.00005	0.250
Flavones	-0.01341	±	0.02265	0.554	-0.01604	±	0.02250	0.476	-0.01633	±	0.02250	0.468	-0.01346	±	0.02252	0.550
Flavonols	0.00099	±	0.00112	0.377	0.00088	±	0.00111	0.425	0.00099	±	0.00111	0.372	0.00077	±	0.00111	0.489
Flavonones	-0.00035	±	0.00071	0.618	-0.00055	±	0.00070	0.431	-0.00055	±	0.00070	0.430	-0.00054	±	0.00070	0.435
Flavan-3-ols	0.00007	±	0.00005	0.161	0.00006	±	0.00005	0.191	0.00007	±	0.00005	0.156	0.00006	±	0.00005	0.192
Anthocyanidins	-0.00040	±	0.00063	0.519	-0.00053	±	0.00063	0.401	-0.00052	±	0.00063	0.402	-0.00046	±	0.00063	0.458

Abbreviations: TMT, Trail Making Test; SE, Standard Error.

[a]Associations are reported for a 10-unit increment in visit 1 flavonoid intake.

[b]Basic Model includes fixed effects for visit 1 flavonoid inake, time, and visit 1 flavonoid intake*time.

[c]Demographic Model is the Basic Model adjusted for visit 1 age in years, age-squared, sex, race, poverty status, education in years, and Wide Range Achievement Test (WRAT) scores.

[d]Lifestyle Model is the Demographic Model adjusted for current smoking status, current drug use, and total energy intake at visit 1.

[e]Clinical Model is the Demographic Model adjusted for diabetes, hypertension, high cholesterol, the Center for Epidemiologic Studies Depression Scale (CES-D), and body mass index (BMI).

Table S4. Association[a] between visit 1 flavonoid intake and ln(TMT-A) for White study participants 50+ years, HANDLS 2004-2020.

	Basic Model[b]				Demographic Model[c]				Lifestyle Model[d]				Clinical Model[e]			
	$\hat{\beta}$	±	SE	p-value	$\hat{\beta}$	±	SE	p-value	$\hat{\beta}$	±	SE	p-value	$\hat{\beta}$	±	SE	p-value
Flavonoid Main Effect: Association between visit 1 flavonoid intake and visit 1 ln(TMT-A)																
Total Flavonoids	-0.00042	±	0.00036	0.244	-0.00023	±	0.00034	0.498	-0.00018	±	0.00034	0.595	-0.00028	±	0.00035	0.419
Flavones	-0.03899	±	0.17968	0.828	0.08152	±	0.17650	0.644	0.13760	±	0.17807	0.440	0.09815	±	0.17540	0.576
Flavonols	-0.01047	±	0.00919	0.255	-0.00488	±	0.00889	0.583	-0.00217	±	0.00895	0.809	-0.00464	±	0.00888	0.602
Flavonones	-0.01180	±	0.00844	0.163	-0.00834	±	0.00830	0.316	-0.00615	±	0.00835	0.462	-0.00856	±	0.00827	0.300
Flavan-3-ols	-0.00037	±	0.00037	0.313	-0.00021	±	0.00036	0.554	-0.00017	±	0.00036	0.636	-0.00026	±	0.00036	0.461
Anthocyanidins	-0.01384	±	0.00755	0.068	-0.00856	±	0.00745	0.251	-0.00710	±	0.00749	0.343	-0.00806	±	0.00742	0.278
*Flavonoid*Time Interaction: Association between visit 1 flavonoid intake and change in ln(TMT-A) over time*																
Total Flavonoids	0.00005	±	0.00005	0.357	0.00005	±	0.00005	0.364	0.00005	±	0.00005	0.333	0.00005	±	0.00005	0.288
Flavones	-0.03308	±	0.02759	0.231	-0.03358	±	0.02743	0.222	-0.03229	±	0.02749	0.240	-0.03273	±	0.02739	0.232
Flavonols	0.00070	±	0.00134	0.604	0.00066	±	0.00133	0.619	0.00073	±	0.00133	0.586	0.00071	±	0.00133	0.592
Flavonones	0.00109	±	0.00138	0.430	0.00095	±	0.00136	0.489	0.00089	±	0.00136	0.512	0.00069	±	0.00136	0.610
Flavan-3-ols	0.00005	±	0.00005	0.353	0.00005	±	0.00005	0.354	0.00005	±	0.00005	0.321	0.00006	±	0.00005	0.281
Anthocyanidins	-0.00002	±	0.00076	0.983	-0.00016	±	0.00076	0.832	-0.00018	±	0.00076	0.808	-0.00009	±	0.00076	0.905

Abbreviations: TMT, Trail Making Test; SE, Standard Error.

[a]Associations are reported for a 10-unit increment in visit 1 flavonoid intake.

[b]Basic Model includes fixed effects for visit 1 flavonoid inake, time, and visit 1 flavonoid intake*time.

[c]Demographic Model is the Basic Model adjusted for visit 1 age in years, age-squared, sex, race, poverty status, education in years, and Wide Range Achievement Test (WRAT) scores.

[d]Lifestyle Model is the Demographic Model adjusted for current smoking status, current drug use, and total energy intake at visit 1.

[e]Clinical Model is the Demographic Model adjusted for diabetes, hypertension, high cholesterol, the Center for Epidemiologic Studies Depression Scale (CES-D), and body mass index (BMI).

Table S5. Association[a] between visit 1 flavonoid intake and ln(TMT-A) for African American study participants 50+ years, HANDLS 2004-2020.

	Basic Model[b]		Demographic Model[c]		Lifestyle Model[d]		Clinical Model[e]	
	$\hat{\beta}$ ± SE	p-value	$\hat{\beta}$ ± SE	p-value	$\hat{\beta}$ ± SE	p-value	$\hat{\beta}$ ± SE	p-value
Flavonoid Main Effect: Association between visit 1 flavonoid intake and visit 1 ln(TMT-A)								
Total Flavonoids	-0.00023 ± 0.00081	0.773	-0.00011 ± 0.00078	0.884	0.00006 ± 0.00078	0.940	0.00007 ± 0.00077	0.929
Flavones	-0.11293 ± 0.30209	0.709	0.16562 ± 0.29551	0.575	0.28540 ± 0.29717	0.337	0.20953 ± 0.29352	0.475
Flavonols	-0.00766 ± 0.01382	0.580	-0.00484 ± 0.01342	0.718	0.00794 ± 0.01410	0.573	-0.00206 ± 0.01330	0.877
Flavonones	-0.00279 ± 0.00682	0.682	-0.00049 ± 0.00654	0.941	0.00177 ± 0.00666	0.790	-0.00012 ± 0.00649	0.986
Flavan-3-ols	-0.00022 ± 0.00085	0.801	-0.00019 ± 0.00082	0.816	-0.00012 ± 0.00081	0.886	0.00000 ± 0.00081	0.999
Anthocyanidins	-0.00163 ± 0.01008	0.872	0.00625 ± 0.00968	0.518	0.01138 ± 0.00980	0.246	0.00572 ± 0.00962	0.552
*Flavonoid*Time Interaction: Association between visit 1 flavonoid intake and change in ln(TMT-A) over time*								
Total Flavonoids	0.00010 ± 0.00010	0.333	0.00009 ± 0.00010	0.360	0.00011 ± 0.00010	0.294	0.00008 ± 0.00010	0.429
Flavones	0.01800 ± 0.03831	0.639	0.01241 ± 0.03822	0.746	0.00931 ± 0.03820	0.808	0.01774 ± 0.03831	0.643
Flavonols	0.00164 ± 0.00195	0.403	0.00165 ± 0.00195	0.397	0.00183 ± 0.00195	0.348	0.00142 ± 0.00195	0.464
Flavonones	-0.00109 ± 0.00085	0.199	-0.00115 ± 0.00084	0.171	-0.00113 ± 0.00084	0.178	-0.00117 ± 0.00084	0.165
Flavan-3-ols	0.00014 ± 0.00011	0.210	0.00013 ± 0.00011	0.227	0.00014 ± 0.00011	0.183	0.00012 ± 0.00011	0.284
Anthocyanidins	-0.00099 ± 0.00109	0.366	-0.00107 ± 0.00109	0.328	-0.00098 ± 0.00109	0.366	-0.00092 ± 0.00109	0.397

Abbreviations: TMT, Trail Making Test; SE, Standard Error.

[a]Associations are reported for a 10-unit increment in visit 1 flavonoid intake.

[b]Basic Model includes fixed effects for visit 1 flavonoid intake, time, and visit 1 flavonoid intake*time.

[c]Demographic Model is the Basic Model adjusted for visit 1 age in years, age-squared, sex, race, poverty status, education in years, and Wide Range Achievement Test (WRAT) scores.

[d]Lifestyle Model is the Demographic Model adjusted for current smoking status, current drug use, and total energy intake at visit 1.

[e]Clinical Model is the Demographic Model adjusted for diabetes, hypertension, high cholesterol, the Center for Epidemiologic Studies Depression Scale (CES-D), and body mass index (BMI).

Table S6. Association[a] between visit 1 flavonoid intake and ln(TMT-B) for all study participants 50+ years, HANDLS 2004-2020.

	**Basic Model[b]**				**Demographic Model[c]**				**Lifestyle Model[d]**				**Clinical Model[e]**			
	$\hat{\beta}$	±	**SE**	**p-value**	$\hat{\beta}$	±	**SE**	**p-value**	$\hat{\beta}$	±	**SE**	**p-value**	$\hat{\beta}$	±	**SE**	**p-value**
Flavonoid Main Effect: Association between visit 1 flavonoid intake and visit 1 ln(TMT-B)																
Total Flavonoids	-0.00104	±	0.00057	0.067	0.00033	±	0.00049	0.504	0.00039	±	0.00049	0.431	0.00021	±	0.00048	0.658
Flavones	-0.78640	±	0.26593	0.003	0.10325	±	0.23446	0.660	0.18130	±	0.23660	0.444	0.16116	±	0.23160	0.487
Flavonols	-0.02973	±	0.01322	0.025	0.00792	±	0.01150	0.491	0.01356	±	0.01177	0.249	0.00771	±	0.01134	0.497
Flavonones	0.00316	±	0.00926	0.733	0.01071	±	0.00796	0.178	0.01370	±	0.00807	0.090	0.01253	±	0.00785	0.111
Flavan-3-ols	-0.00095	±	0.00059	0.108	0.00030	±	0.00051	0.550	0.00034	±	0.00051	0.508	0.00016	±	0.00050	0.745
Anthocyanidins	-0.03676	±	0.01057	0.001	-0.00436	±	0.00922	0.636	-0.00143	±	0.00932	0.878	-0.00100	±	0.00912	0.913
*Flavonoid*Time Interaction: Association between visit 1 flavonoid intake and change in ln(TMT-B) over time*																
Total Flavonoids	0.00010	±	0.00006	0.122	0.00009	±	0.00006	0.166	0.00009	±	0.00006	0.156	0.00008	±	0.00006	0.199
Flavones	0.00398	±	0.03092	0.898	0.00473	±	0.03071	0.878	0.00396	±	0.03075	0.898	0.00429	±	0.03069	0.889
Flavonols	0.00190	±	0.00152	0.212	0.00175	±	0.00151	0.247	0.00180	±	0.00151	0.234	0.00136	±	0.00151	0.366
Flavonones	-0.00077	±	0.00097	0.426	-0.00102	±	0.00096	0.288	-0.00103	±	0.00096	0.282	-0.00130	±	0.00096	0.175
Flavan-3-ols	0.00011	±	0.00007	0.104	0.00010	±	0.00007	0.144	0.00010	±	0.00007	0.135	0.00009	±	0.00007	0.163
Anthocyanidins	0.00014	±	0.00085	0.873	0.00001	±	0.00085	0.989	-0.00003	±	0.00085	0.976	-0.00007	±	0.00085	0.930

Abbreviations: TMT, Trail Making Test; SE, Standard Error.

[a]Associations are reported for a 10-unit increment in visit 1 flavonoid intake.

[b]Basic Model includes fixed effects for visit 1 flavonoid intake, time, and visit 1 flavonoid intake*time.

[c]Demographic Model is the Basic Model adjusted for visit 1 age in years, age-squared, sex, race, poverty status, education in years, and Wide Range Achievement Test (WRAT) scores.

[d]Lifestyle Model is the Demographic Model adjusted for current smoking status, current drug use, and total energy intake at visit 1.

[e]Clinical Model is the Demographic Model adjusted for diabetes, hypertension, high cholesterol, the Center for Epidemiologic Studies Depression Scale (CES-D), and body mass index (BMI).

Table S7. Association[a] between visit 1 flavonoid intake and ln(TMT-B) for White study participants 50+ years, HANDLS 2004-2020.

	**Basic Model[b]**			**Demographic Model[c]**			**Lifestyle Model[d]**			**Clinical Model[e]**		
	$\hat{\beta}$	± SE	p-value	$\hat{\beta}$	± SE	p-value	$\hat{\beta}$	± SE	p-value	$\hat{\beta}$	± SE	p-value
Flavonoid Main Effect: Association between visit 1 flavonoid intake and visit 1 ln(TMT-B)												
Total Flavonoids	-0.00034	± 0.00053	0.521	-0.00002	± 0.00049	0.966	0.00001	± 0.00049	0.980	-0.00013	± 0.00049	0.795
Flavones	-0.58736	± 0.26390	0.027	-0.22680	± 0.24842	0.362	-0.17044	± 0.25119	0.497	-0.18233	± 0.24680	0.460
Flavonols	-0.01459	± 0.01362	0.285	0.00044	± 0.01254	0.972	0.00288	± 0.01265	0.820	-0.00011	± 0.01252	0.993
Flavonones	-0.02005	± 0.01242	0.107	-0.00091	± 0.01168	0.938	0.00137	± 0.01177	0.908	0.00050	± 0.01163	0.966
Flavan-3-ols	-0.00020	± 0.00055	0.716	0.00003	± 0.00050	0.958	0.00005	± 0.00050	0.923	-0.00009	± 0.00051	0.855
Anthocyanidins	-0.03814	± 0.01114	0.001	-0.01931	± 0.01055	0.068	-0.01750	± 0.01062	0.099	-0.01758	± 0.01051	0.094
*Flavonoid*Time Interaction: Association between visit 1 flavonoid intake and change in ln(TMT-B) over time*												
Total Flavonoids	0.00011	± 0.00007	0.094	0.00010	± 0.00007	0.127	0.00010	± 0.00007	0.123	0.00010	± 0.00007	0.142
Flavones	0.02177	± 0.03544	0.539	0.02641	± 0.03533	0.455	0.02748	± 0.03542	0.438	0.02591	± 0.03535	0.464
Flavonols	0.00279	± 0.00171	0.105	0.00255	± 0.00171	0.136	0.00260	± 0.00171	0.129	0.00225	± 0.00171	0.190
Flavonones	0.00066	± 0.00179	0.714	0.00072	± 0.00177	0.683	0.00072	± 0.00178	0.685	0.00037	± 0.00178	0.833
Flavan-3-ols	0.00011	± 0.00007	0.100	0.00010	± 0.00007	0.133	0.00010	± 0.00007	0.128	0.00010	± 0.00007	0.146
Anthocyanidins	0.00029	± 0.00097	0.765	0.00013	± 0.00097	0.890	0.00009	± 0.00097	0.922	0.00017	± 0.00097	0.863

Abbreviations: TMT, Trail Making Test; SE, Standard Error.

[a]Associations are reported for a 10-unit increment in visit 1 flavonoid intake.

[b]Basic Model includes fixed effects for visit 1 flavonoid intake, time, and visit 1 flavonoid intake*time.

[c]Demographic Model is the Basic Model adjusted for visit 1 age in years, age-squared, sex, race, poverty status, education in years, and Wide Range Achievement Test (WRAT) scores.

[d]Lifestyle Model is the Demographic Model adjusted for current smoking status, current drug use, and total energy intake at visit 1.

[e]Clinical Model is the Demographic Model adjusted for diabetes, hypertension, high cholesterol, the Center for Epidemiologic Studies Depression Scale (CES-D), and body mass index (BMI).

Table S8. Association[a] between visit 1 flavonoid intake and ln(TMT-B) for African American study participants 50+ years, HANDLS 2004-2020.

	Basic Model[b]				Demographic Model[c]				Lifestyle Model[d]				Clinical Model[e]			
	$\hat{\beta}$	±	SE	p-value	$\hat{\beta}$	±	SE	p-value	$\hat{\beta}$	±	SE	p-value	$\hat{\beta}$	±	SE	p-value
Flavonoid Main Effect: Association between visit 1 flavonoid intake and visit 1 ln(TMT-B)																
Total Flavonoids	0.00170	±	0.00142	0.232	0.00225	±	0.00128	0.079	0.00241	±	0.00129	0.061	0.00253	±	0.00126	0.045
Flavones	0.03019	±	0.53028	0.955	0.95456	±	0.48897	0.051	1.08187	±	0.49397	0.029	1.09100	±	0.48170	0.024
Flavonols	0.00757	±	0.02422	0.755	0.02769	±	0.02215	0.211	0.04114	±	0.02352	0.080	0.03121	±	0.02180	0.152
Flavonones	0.00911	±	0.01201	0.449	0.01650	±	0.01084	0.128	0.01928	±	0.01106	0.082	0.01845	±	0.01068	0.084
Flavan-3-ols	0.00168	±	0.00150	0.263	0.00197	±	0.00135	0.145	0.00203	±	0.00135	0.134	0.00220	±	0.00133	0.098
Anthocyanidins	0.00367	±	0.01790	0.838	0.02059	±	0.01617	0.203	0.02567	±	0.01647	0.119	0.02442	±	0.01595	0.126
*Flavonoid*Time Interaction: Association between visit 1 flavonoid intake and change in ln(TMT-B) over time*																
Total Flavonoids	-0.00006	±	0.00015	0.696	-0.00006	±	0.00015	0.696	-0.00005	±	0.00015	0.734	-0.00005	±	0.00015	0.754
Flavones	-0.05359	±	0.05422	0.323	-0.05945	±	0.05383	0.269	-0.06266	±	0.05395	0.245	-0.06153	±	0.05392	0.254
Flavonols	-0.00126	±	0.00277	0.650	-0.00112	±	0.00275	0.684	-0.00103	±	0.00275	0.710	-0.00127	±	0.00275	0.645
Flavonones	-0.00124	±	0.00120	0.300	-0.00133	±	0.00119	0.263	-0.00134	±	0.00119	0.261	-0.00171	±	0.00119	0.150
Flavan-3-ols	-0.00003	±	0.00016	0.858	-0.00003	±	0.00016	0.862	-0.00002	±	0.00016	0.902	-0.00001	±	0.00016	0.973
Anthocyanidins	-0.00091	±	0.00153	0.554	-0.00099	±	0.00152	0.515	-0.00097	±	0.00152	0.522	-0.00125	±	0.00152	0.413

Abbreviations: TMT, Trail Making Test; SE, Standard Error.

[a]Associations are reported for a 10-unit increment in visit 1 flavonoid intake.

[b]Basic Model includes fixed effects for visit 1 flavonoid intake, time, and visit 1 flavonoid intake*time.

[c]Demographic Model is the Basic Model adjusted for visit 1 age in years, age-squared, sex, race, poverty status, education in years, and Wide Range Achievement Test (WRAT) scores.

[d]Lifestyle Model is the Demographic Model adjusted for current smoking status, current drug use, and total energy intake at visit 1.

[e]Clinical Model is the Demographic Model adjusted for diabetes, hypertension, high cholesterol, the Center for Epidemiologic Studies Depression Scale (CES-D), and body mass index (BMI).

Table S9. Association[a] between visit 1 flavonoid intake and ln(TMT-A) for all study participants without MMSE exclusion, HANDLS 2004-2020.

	**Basic Model[b]**				**Demographic Model[c]**				**Lifestyle Model[d]**				**Clinical Model[e]**			
	$\hat{\beta}$	±	SE	p-value	$\hat{\beta}$	±	SE	p-value	$\hat{\beta}$	±	SE	p-value	$\hat{\beta}$	±	SE	p-value
Flavonoid Main Effect: Association between visit 1 flavonoid intake and visit 1 ln(TMT-A)																
Total Flavonoids	-0.00050	±	0.00020	0.012	0.00000	±	0.00018	0.983	0.00004	±	0.00018	0.807	-0.00005	±	0.00017	0.756
Flavones	-0.30914	±	0.10076	0.002	0.03359	±	0.09095	0.712	0.08347	±	0.09151	0.362	0.04103	±	0.09026	0.649
Flavonols	-0.01502	±	0.00485	0.002	-0.00236	±	0.00435	0.587	0.00142	±	0.00442	0.748	-0.00309	±	0.00431	0.474
Flavonones	-0.00237	±	0.00363	0.513	-0.00508	±	0.00322	0.115	-0.00354	±	0.00324	0.274	-0.00425	±	0.00320	0.184
Flavan-3-ols	-0.00047	±	0.00020	0.022	0.00003	±	0.00018	0.872	0.00006	±	0.00018	0.758	-0.00004	±	0.00018	0.840
Anthocyanidins	-0.01706	±	0.00507	0.001	-0.00492	±	0.00457	0.281	-0.00292	±	0.00458	0.523	-0.00376	±	0.00454	0.408
*Flavonoid*Time Interaction: Association between visit 1 flavonoid intake and change in ln(TMT-A) over time*																
Total Flavonoids	0.00000	±	0.00002	0.995	-0.00001	±	0.00002	0.810	-0.00001	±	0.00002	0.827	-0.00001	±	0.00002	0.818
Flavones	-0.01676	±	0.01192	0.160	-0.01989	±	0.01182	0.092	-0.02068	±	0.01183	0.080	-0.02001	±	0.01182	0.091
Flavonols	0.00022	±	0.00059	0.708	0.00012	±	0.00059	0.845	0.00015	±	0.00059	0.804	0.00008	±	0.00059	0.886
Flavonones	-0.00008	±	0.00043	0.851	-0.00019	±	0.00043	0.657	-0.00021	±	0.00043	0.628	-0.00015	±	0.00043	0.724
Flavan-3-ols	0.00000	±	0.00002	0.965	0.00000	±	0.00002	0.855	0.00000	±	0.00002	0.870	0.00000	±	0.00002	0.855
Anthocyanidins	-0.00001	±	0.00050	0.988	-0.00017	±	0.00050	0.737	-0.00017	±	0.00050	0.737	-0.00012	±	0.00050	0.809

Abbreviations: TMT, Trail Making Test; MMSE, Mini-Mental State Exam; SE, Standard Error.

[a]Associations are reported for a 10-unit increment in visit 1 flavonoid intake.

[b]Basic Model includes fixed effects for visit 1 flavonoid intake, time, and visit 1 flavonoid intake*time.

[c]Demographic Model is the Basic Model adjusted for visit 1 age in years, age-squared, sex, race, poverty status, education in years, and Wide Range Achievement Test (WRAT) scores.

[d]Lifestyle Model is the Demographic Model adjusted for current smoking status, current drug use, and total energy intake at visit 1.

[e]Clinical Model is the Demographic Model adjusted for diabetes, hypertension, high cholesterol, the Center for Epidemiologic Studies Depression Scale (CES-D), and body mass index (BMI).

Table S10. Association[a] between visit 1 flavonoid intake and ln(TMT-A) for White study participants without MMSE exclusion, HANDLS 2004-2020.

	**Basic Model[b]**				**Demographic Model[c]**				**Lifestyle Model[d]**				**Clinical Model[e]**			
	$\hat{\boldsymbol{\beta}}$	**±**	**SE**	**p-value**	$\hat{\boldsymbol{\beta}}$	**±**	**SE**	**p-value**	$\hat{\boldsymbol{\beta}}$	**±**	**SE**	**p-value**	$\hat{\boldsymbol{\beta}}$	**±**	**SE**	**p-value**
Flavonoid Main Effect: Association between visit 1 flavonoid intake and visit 1 ln(TMT-A)																
Total Flavonoids	-0.00007	±	0.00020	0.727	0.00003	±	0.00018	0.881	0.00005	±	0.00018	0.769	-0.00002	±	0.00018	0.899
Flavones	-0.17236	±	0.11441	0.132	-0.00599	±	0.10708	0.955	0.03532	±	0.10779	0.743	0.00053	±	0.10634	0.996
Flavonols	-0.00455	±	0.00522	0.383	-0.00030	±	0.00480	0.950	0.00179	±	0.00483	0.711	-0.00113	±	0.00477	0.813
Flavonones	-0.00215	±	0.00578	0.710	-0.00299	±	0.00535	0.576	-0.00162	±	0.00537	0.763	-0.00233	±	0.00533	0.663
Flavan-3-ols	-0.00004	±	0.00020	0.843	0.00004	±	0.00019	0.820	0.00006	±	0.00019	0.733	-0.00002	±	0.00019	0.934
Anthocyanidins	-0.01735	±	0.00582	0.003	-0.00887	±	0.00554	0.109	-0.00780	±	0.00555	0.160	-0.00870	±	0.00552	0.115
*Flavonoid*Time Interaction: Association between visit 1 flavonoid intake and change in ln(TMT-A) over time*																
Total Flavonoids	-0.00001	±	0.00002	0.768	-0.00001	±	0.00002	0.666	-0.00001	±	0.00002	0.663	0.00000	±	0.00002	0.838
Flavones	-0.03119	±	0.01472	0.034	-0.03098	±	0.01470	0.035	-0.03108	±	0.01472	0.035	-0.02989	±	0.01467	0.042
Flavonols	-0.00034	±	0.00064	0.596	-0.00040	±	0.00064	0.535	-0.00038	±	0.00064	0.556	-0.00027	±	0.00064	0.673
Flavonones	0.00098	±	0.00083	0.237	0.00096	±	0.00082	0.240	0.00090	±	0.00082	0.270	0.00094	±	0.00082	0.249
Flavan-3-ols	-0.00001	±	0.00002	0.772	-0.00001	±	0.00002	0.675	-0.00001	±	0.00002	0.672	0.00000	±	0.00002	0.842
Anthocyanidins	-0.00006	±	0.00060	0.915	-0.00026	±	0.00060	0.667	-0.00027	±	0.00060	0.657	-0.00020	±	0.00060	0.732

Abbreviations: TMT, Trail Making Test; MMSE, Mini-Mental State Exam; SE, Standard Error.

[a]Associations are reported for a 10-unit increment in visit 1 flavonoid intake.

[b]Basic Model includes fixed effects for visit 1 flavonoid intake, time, and visit 1 flavonoid intake*time.

[c]Demographic Model is the Basic Model adjusted for visit 1 age in years, age-squared, sex, race, poverty status, education in years, and Wide Range Achievement Test (WRAT) scores.

[d]Lifestyle Model is the Demographic Model adjusted for current smoking status, current drug use, and total energy intake at visit 1.

[e]Clinical Model is the Demographic Model adjusted for diabetes, hypertension, high cholesterol, the Center for Epidemiologic Studies Depression Scale (CES-D), and body mass index (BMI).

Table S11. Association[a] between visit 1 flavonoid intake and ln(TMT-A) for African American study participants without MMSE exclusion, HANDLS 2004-2020.

	Basic Model[b]				Demographic Model[c]				Lifestyle Model[d]				Clinical Model[e]			
	$\hat{\beta}$	±	SE	p-value	$\hat{\beta}$	±	SE	p-value	$\hat{\beta}$	±	SE	p-value	$\hat{\beta}$	±	SE	p-value
Flavonoid Main Effect: Association between visit 1 flavonoid intake and visit 1 ln(TMT-A)																
Total Flavonoids	-0.00057	±	0.00048	0.230	-0.00020	±	0.00043	0.647	-0.00010	±	0.00043	0.817	-0.00017	±	0.00043	0.700
Flavones	-0.14196	±	0.17133	0.408	0.07716	±	0.15538	0.620	0.13957	±	0.15657	0.373	0.07875	±	0.15417	0.610
Flavonols	-0.01430	±	0.00922	0.121	-0.00888	±	0.00841	0.291	-0.00149	±	0.00878	0.866	-0.00818	±	0.00835	0.327
Flavonones	-0.00761	±	0.00452	0.092	-0.00536	±	0.00407	0.187	-0.00394	±	0.00410	0.337	-0.00500	±	0.00404	0.216
Flavan-3-ols	-0.00048	±	0.00050	0.338	-0.00014	±	0.00045	0.760	-0.00008	±	0.00045	0.854	-0.00011	±	0.00045	0.803
Anthocyanidins	-0.00272	±	0.00834	0.745	0.00011	±	0.00751	0.988	0.00341	±	0.00757	0.653	0.00070	±	0.00746	0.925
*Flavonoid*Time Interaction: Association between visit 1 flavonoid intake and change in ln(TMT-A) over time*																
Total Flavonoids	0.00004	±	0.00006	0.498	0.00003	±	0.00006	0.615	0.00003	±	0.00006	0.595	0.00002	±	0.00006	0.770
Flavones	-0.00290	±	0.01847	0.875	-0.00817	±	0.01828	0.655	-0.01010	±	0.01832	0.581	-0.00966	±	0.01830	0.598
Flavonols	0.00158	±	0.00113	0.164	0.00146	±	0.00112	0.193	0.00147	±	0.00112	0.188	0.00119	±	0.00112	0.287
Flavonones	-0.00037	±	0.00053	0.484	-0.00054	±	0.00052	0.297	-0.00055	±	0.00052	0.289	-0.00050	±	0.00052	0.331
Flavan-3-ols	0.00004	±	0.00006	0.464	0.00003	±	0.00006	0.553	0.00004	±	0.00006	0.535	0.00002	±	0.00006	0.714
Anthocyanidins	0.00005	±	0.00081	0.952	0.00009	±	0.00080	0.911	0.00008	±	0.00080	0.916	0.00011	±	0.00080	0.889

Abbreviations: TMT, Trail Making Test; MMSE, Mini-Mental State Exam; SE, Standard Error.

[a]Associations are reported for a 10-unit increment in visit 1 flavonoid intake.

[b]Basic Model includes fixed effects for visit 1 flavonoid intake, time, and visit 1 flavonoid intake*time.

[c]Demographic Model is the Basic Model adjusted for visit 1 age in years, age-squared, sex, race, poverty status, education in years, and Wide Range Achievement Test (WRAT) scores.

[d]Lifestyle Model is the Demographic Model adjusted for current smoking status, current drug use, and total energy intake at visit 1.

[e]Clinical Model is the Demographic Model adjusted for diabetes, hypertension, high cholesterol, the Center for Epidemiologic Studies Depression Scale (CES-D), and body mass index (BMI).

Table S12. Association[a] between visit 1 flavonoid intake and ln(TMT-B) for all study participants without MMSE exclusion, HANDLS 2004-2020.

	Basic Model[b]				Demographic Model[c]				Lifestyle Model[d]				Clinical Model[e]			
	$\widehat{\beta}$	±	SE	p-value	$\widehat{\beta}$	±	SE	p-value	$\widehat{\beta}$	±	SE	p-value	$\widehat{\beta}$	±	SE	p-value
Flavonoid Main Effect: Association between visit 1 flavonoid intake and visit 1 ln(TMT-B)																
Total Flavonoids	-0.00120	±	0.00032	<0.001	-0.00024	±	0.00027	0.368	-0.00019	±	0.00027	0.477	-0.00029	±	0.00026	0.268
Flavones	-1.03293	±	0.16506	<0.001	-0.21236	±	0.13782	0.123	-0.14283	±	0.13888	0.304	-0.18034	±	0.13612	0.185
Flavonols	-0.03209	±	0.00798	<0.001	-0.00462	±	0.00658	0.483	0.00083	±	0.00671	0.901	-0.00473	±	0.00650	0.467
Flavonones	0.00618	±	0.00597	0.301	0.00310	±	0.00487	0.524	0.00556	±	0.00491	0.257	0.00492	±	0.00482	0.307
Flavan-3-ols	-0.00117	±	0.00034	0.001	-0.00025	±	0.00028	0.370	-0.00022	±	0.00028	0.434	-0.00031	±	0.00027	0.249
Anthocyanidins	-0.03891	±	0.00833	<0.001	-0.00498	±	0.00693	0.472	-0.00194	±	0.00696	0.780	-0.00205	±	0.00685	0.765
*Flavonoid*Time Interaction: Association between visit 1 flavonoid intake and change in ln(TMT-B) over time*																
Total Flavonoids	0.00006	±	0.00003	0.051	0.00005	±	0.00003	0.096	0.00005	±	0.00003	0.092	0.00005	±	0.00003	0.122
Flavones	0.01908	±	0.01605	0.235	0.01657	±	0.01588	0.297	0.01560	±	0.01590	0.326	0.01601	±	0.01587	0.313
Flavonols	0.00158	±	0.00080	0.049	0.00141	±	0.00079	0.076	0.00144	±	0.00079	0.070	0.00121	±	0.00079	0.128
Flavonones	-0.00018	±	0.00058	0.752	-0.00033	±	0.00057	0.558	-0.00036	±	0.00057	0.534	-0.00037	±	0.00057	0.518
Flavan-3-ols	0.00007	±	0.00003	0.053	0.00006	±	0.00003	0.097	0.00006	±	0.00003	0.094	0.00005	±	0.00003	0.122
Anthocyanidins	0.00056	±	0.00068	0.411	0.00038	±	0.00067	0.568	0.00037	±	0.00067	0.583	0.00037	±	0.00066	0.581

Abbreviations: TMT, Trail Making Test; MMSE, Mini-Mental State Exam; SE, Standard Error.

[a]Associations are reported for a 10-unit increment in visit 1 flavonoid intake.

[b]Basic Model includes fixed effects for visit 1 flavonoid intake, time, and visit 1 flavonoid intake*time.

[c]Demographic Model is the Basic Model adjusted for visit 1 age in years, age-squared, sex, race, poverty status, education in years, and Wide Range Achievement Test (WRAT) scores.

[d]Lifestyle Model is the Demographic Model adjusted for current smoking status, current drug use, and total energy intake at visit 1.

[e]Clinical Model is the Demographic Model adjusted for diabetes, hypertension, high cholesterol, the Center for Epidemiologic Studies Depression Scale (CES-D), and body mass index (BMI).

Table S13. Association[a] between visit 1 flavonoid intake and ln(TMT-B) for White study participants without MMSE exclusion, HANDLS 2004-2020.

	Basic Model[b]				Demographic Model[c]				Lifestyle Model[d]				Clinical Model[e]			
	$\hat{\beta}$	±	SE	p-value	$\hat{\beta}$	±	SE	p-value	$\hat{\beta}$	±	SE	p-value	$\hat{\beta}$	±	SE	p-value
Flavonoid Main Effect: Association between visit 1 flavonoid intake and visit 1 ln(TMT-B)																
Total Flavonoids	-0.00037	±	0.00033	0.261	-0.00024	±	0.00027	0.371	-0.00021	±	0.00027	0.426	-0.00030	±	0.00027	0.265
Flavones	-0.85911	±	0.18808	<0.001	-0.38499	±	0.15796	0.015	-0.33718	±	0.15939	0.034	-0.35813	±	0.15662	0.022
Flavonols	-0.01578	±	0.00866	0.069	-0.00517	±	0.00710	0.467	-0.00265	±	0.00717	0.712	-0.00570	±	0.00705	0.418
Flavonones	-0.01029	±	0.00955	0.282	-0.00692	±	0.00789	0.381	-0.00515	±	0.00793	0.516	-0.00413	±	0.00785	0.599
Flavan-3-ols	-0.00028	±	0.00034	0.407	-0.00021	±	0.00028	0.447	-0.00019	±	0.00028	0.491	-0.00028	±	0.00028	0.314
Anthocyanidins	-0.05140	±	0.00958	<0.001	-0.02115	±	0.00820	0.010	-0.01957	±	0.00824	0.018	-0.01916	±	0.00817	0.019
*Flavonoid*Time Interaction: Association between visit 1 flavonoid intake and change in ln(TMT-B) over time*																
Total Flavonoids	0.00005	±	0.00003	0.128	0.00004	±	0.00003	0.241	0.00004	±	0.00003	0.235	0.00004	±	0.00003	0.247
Flavones	0.02823	±	0.01963	0.151	0.02640	±	0.01947	0.175	0.02622	±	0.01951	0.179	0.02872	±	0.01941	0.139
Flavonols	0.00150	±	0.00086	0.083	0.00117	±	0.00085	0.170	0.00120	±	0.00085	0.160	0.00110	±	0.00085	0.197
Flavonones	0.00086	±	0.00112	0.441	0.00081	±	0.00110	0.461	0.00079	±	0.00110	0.474	0.00074	±	0.00109	0.501
Flavan-3-ols	0.00005	±	0.00003	0.153	0.00004	±	0.00003	0.278	0.00004	±	0.00003	0.271	0.00004	±	0.00003	0.282
Anthocyanidins	0.00131	±	0.00081	0.105	0.00108	±	0.00080	0.174	0.00106	±	0.00080	0.186	0.00106	±	0.00079	0.184

Abbreviations: TMT, Trail Making Test; MMSE, Mini-Mental State Exam; SE, Standard Error.

[a]Associations are reported for a 10-unit increment in visit 1 flavonoid intake.

[b]Basic Model includes fixed effects for visit 1 flavonoid intake, time, and visit 1 flavonoid intake*time.

[c]Demographic Model is the Basic Model adjusted for visit 1 age in years, age-squared, sex, race, poverty status, education in years, and Wide Range Achievement Test (WRAT) scores.

[d]Lifestyle Model is the Demographic Model adjusted for current smoking status, current drug use, and total energy intake at visit 1.

[e]Clinical Model is the Demographic Model adjusted for diabetes, hypertension, high cholesterol, the Center for Epidemiologic Studies Depression Scale (CES-D), and body mass index (BMI).

Table S14. Association[a] between visit 1 flavonoid intake and ln(TMT-B) for African American study participants without MMSE exclusion, HANDLS 2004-2020.

	Basic Model[b]				Demographic Model[c]				Lifestyle Model[d]				Clinical Model[e]			
	$\hat{\beta}$	±	SE	p-value	$\hat{\beta}$	±	SE	p-value	$\hat{\beta}$	±	SE	p-value	$\hat{\beta}$	±	SE	p-value
Flavonoid Main Effect: Association between visit 1 flavonoid intake and visit 1 ln(TMT-B)																
Total Flavonoids	-0.00090	±	0.00076	0.241	-0.00001	±	0.00066	0.993	0.00016	±	0.00066	0.813	0.00012	±	0.00065	0.858
Flavones	-0.51534	±	0.27456	0.061	0.05560	±	0.23864	0.816	0.15961	±	0.24053	0.507	0.08918	±	0.23524	0.705
Flavonols	-0.01469	±	0.01473	0.319	0.00047	±	0.01286	0.971	0.01354	±	0.01345	0.314	0.00277	±	0.01267	0.827
Flavonones	0.00269	±	0.00725	0.710	0.00751	±	0.00624	0.228	0.01040	±	0.00629	0.098	0.00839	±	0.00615	0.172
Flavan-3-ols	-0.00097	±	0.00080	0.224	-0.00017	±	0.00069	0.800	-0.00008	±	0.00069	0.901	-0.00007	±	0.00068	0.916
Anthocyanidins	0.01040	±	0.01333	0.435	0.01971	±	0.01148	0.086	0.02555	±	0.01157	0.027	0.02211	±	0.01132	0.051
*Flavonoid*Time Interaction: Association between visit 1 flavonoid intake and change in ln(TMT-B) over time*																
Total Flavonoids	0.00007	±	0.00008	0.386	0.00006	±	0.00008	0.415	0.00006	±	0.00008	0.401	0.00005	±	0.00008	0.537
Flavones	-0.00244	±	0.02504	0.922	-0.00501	±	0.02480	0.840	-0.00799	±	0.02485	0.748	-0.00864	±	0.02486	0.728
Flavonols	0.00104	±	0.00154	0.499	0.00113	±	0.00152	0.457	0.00117	±	0.00152	0.444	0.00069	±	0.00152	0.653
Flavonones	-0.00028	±	0.00070	0.689	-0.00048	±	0.00070	0.489	-0.00050	±	0.00070	0.468	-0.00050	±	0.00070	0.471
Flavan-3-ols	0.00008	±	0.00008	0.328	0.00008	±	0.00008	0.344	0.00008	±	0.00008	0.331	0.00006	±	0.00008	0.448
Anthocyanidins	-0.00097	±	0.00108	0.368	-0.00094	±	0.00107	0.380	-0.00097	±	0.00107	0.365	-0.00096	±	0.00107	0.368

Abbreviations: TMT, Trail Making Test; MMSE, Mini-Mental State Exam; SE, Standard Error.

[a]Associations are reported for a 10-unit increment in visit 1 flavonoid intake.

[b]Basic Model includes fixed effects for visit 1 flavonoid intake, time, and visit 1 flavonoid intake*time.

[c]Demographic Model is the Basic Model adjusted for visit 1 age in years, age-squared, sex, race, poverty status, education in years, and Wide Range Achievement Test (WRAT) scores.

[d]Lifestyle Model is the Demographic Model adjusted for current smoking status, current drug use, and total energy intake at visit 1.

[e]Clinical Model is the Demographic Model adjusted for diabetes, hypertension, high cholesterol, the Center for Epidemiologic Studies Depression Scale (CES-D), and body mass index (BMI).
